# D-beta-hydroxybutyrate extends lifespan in *C. elegans*

**DOI:** 10.18632/aging.100683

**Published:** 2014-08-07

**Authors:** Clare Edwards, John Canfield, Neil Copes, Muhammad Rehan, David Lipps, Patrick C. Bradshaw

**Affiliations:** Department of Cell Biology, Microbiology, and Molecular Biology, University of South Florida, Tampa, FL 33620, USA

**Keywords:** C. elegans, aging, lifespan, beta-hydroxybutyrate, ketone bodies, mitochondria

## Abstract

The ketone body beta-hydroxybutyrate (βHB) is a histone deacetylase (HDAC) inhibitor and has been shown to be protective in many disease models, but its effects on aging are not well studied. Therefore we determined the effect of βHB supplementation on the lifespan of *C. elegans* nematodes. βHB supplementation extended mean lifespan by approximately 20%. RNAi knockdown of HDACs *hda-2* or *hda-3* also increased lifespan and further prevented βHB-mediated lifespan extension. βHB-mediated lifespan extension required the DAF-16/FOXO and SKN-1/Nrf longevity pathways, the sirtuin SIR-2.1, and the AMP kinase subunit AAK-2. βHB did not extend lifespan in a genetic model of dietary restriction indicating that βHB is likely functioning through a similar mechanism. βHB addition also upregulated βHB dehydrogenase activity and increased oxygen consumption in the worms. RNAi knockdown of F55E10.6, a short chain dehydrogenase and SKN-1 target gene, prevented the increased lifespan and βHB dehydrogenase activity induced by βHB addition, suggesting that F55E10.6 functions as an inducible βHB dehydrogenase. Furthermore, βHB supplementation increased worm thermotolerance and partially prevented glucose toxicity. It also delayed Alzheimer's amyloid-beta toxicity and decreased Parkinson's alpha-synuclein aggregation. The results indicate that D-βHB extends lifespan through inhibiting HDACs and through the activation of conserved stress response pathways.

## INTRODUCTION

Aging leads to a progressive decline of cell and tissue function and is the primary risk factor for many ailments, including the prevalent neurodegenerative disorders Alzheimer's disease (AD) and Parkinson's disease (PD). Mitochondria are the central hub of cellular metabolism and mitochondrial dysfunction, especially in stem cells [1], has been shown to cause the development of premature aging phenotypes in mice [2]. Paradoxically, slight inhibition of mitochondrial respiration can also lead to small increases in reactive oxygen species (ROS) production and extend the lifespan of yeast, *C. elegans*, *Drosophila*, and mice [3-6]. Even in young animals, roughly 0.15% of electrons passing through the mitochondrial electron transport chain (ETC) combine with molecular oxygen to form superoxide [7, 8]. Mitochondrial ROS production increases with age and leads to progressive damage of cellular macromolecules as outlined in the mitochondrial free radical theory of aging [9].

Dietary restriction (DR) increases the lifespan of many organisms including *C. elegans* [10]. As interest in the molecular mechanisms responsible for the effect of DR on lifespan have expanded, so has the discovery of the pathways involved and the search for DR mimetic compounds that promote survival and stress resistance [11, 12]. The ketone body beta-hydroxybutyrate (βHB) has been described as a DR mimetic compound [13], in part because it increases in the plasma during DR and when administered exogenously leads to decreased levels of oxidative stress [14]. In mammals, βHB is produced in the liver, primarily from the catabolic breakdown of fatty acids, and is used as an alternative energy source when blood glucose is low. This is especially important in the brain where only a very limited amount of fatty acid beta-oxidation takes place [15]. In mitochondria βHB is catabolized to acetoacetate by βHB dehydrogenase 1 (BDH1). The reaction is linked to the reduction of NAD to NADH, which fuels mitochondrial ETC complex I. The resulting acetoacetate is catabolized to acetoacetyl-CoA and then to acetyl-CoA, which is metabolized as part of the TCA cycle. The βHB dehydrogenase 2 (BDH2) enzyme localizes to the cytoplasm, but no changes in ketone body metabolism were found in BDH2 knockout mice suggesting BDH2 plays a limited role, if any, in ketone body metabolism [16].

Researchers are investigating DR mimetics not only for their possible lifespan extending capabilities, but also for their potential ability to delay the onset and progression of age-associated diseases such as AD. [17, 18]. Phenotypes of AD brain include extracellular senile plaques containing Aß peptide as well as intracellular neurofibrillary tangles consisting of hyper-phosphorylated tau protein [19]. Intracellular Aß can inhibit mitochondrial ETC complex IV and increase ROS production [20]. βHB has shown some efficacy in the protection against AD-mediated neurodegeneration in animal models and human trials. βHB protected cultured hippocampal neurons from Aß _1–42_ toxicity [21]. βHB or a ketogenic diet has shown mixed effects on disease phenotypes in mouse models of AD. For example in one study, a ketogenic diet lowered Aß levels, but did not affect cognitive impairment [22]. In another study a ketogenic diet improved motor function, but did not affect cognition or tau or Aß pathology [23]. However, a further study found that supplementation of βHB methyl ester to AD mice was able to restore cognitive function and decrease Aß levels, likely due to the fact that the methyl ester is transported through the blood-brain barrier more efficiently than the free acid [24]. There is also evidence for the clinical use of ketone bodies to treat neurodegenerative disorders as oral ingestion of medium chain triglycerides, which are catabolized in part to ketone bodies, increased plasma levels of βHB and led to improved cognitive function in human patients with AD [25].

PD, another aging-associated disorder, is characterized by an accumulation of Lewy bodies in the substantia nigra region of the brain. The alpha-synuclein protein is a major component of Lewy bodies and can also localize to mitochondrial membranes [26] causing decreased ETC complex I activity with an accompanying increase in ROS production [27]. This may be partly responsible for the increased mitochondrial oxidative damage that has been observed in brains from autopsied PD patients [28, 29]. βHB has also been shown to be efficacious in several animal models of PD. Mice treated with βHB showed partial protection against neurodegeneration and motor deficiency induced by the neurotoxin 1-methyl-4-phenyl-1,2,3,6-tetrahydropyridine (MPTP), which induces PD-like symptoms [30]. Additionally, βHB protected cultured neurons from toxicity induced by the structurally related ETC complex I inhibitor 1-methyl-4-phenylpyridinium (MPP(+)) [21]. In human clinical trials, PD patients treated with a ketogenic diet for one month improved their Unified Parkinson's Disease Rating Scale scores by a mean of 43% [31].

Although much is known about the effects of βHB on neurodegenerative and other aging-associated diseases, not much is known about its effects on aging. Moreover, the mechanisms through which βHB are protective are not entirely clear. However, recent evidence suggests that βHB protects against oxidative stress through its action as a class I and class IIa histone deacetylase inhibitor to increase expression of stress response genes such as FoxO3A and MT2 [14]. In this report we determined the effect of βHB on lifespan in *C. elegans* and determined the cytoprotective signaling pathways required for this effect. We then determined the effects of βHB on proteotoxicity in nematode models of AD, PD, and amyotrophic lateral sclerosis (ALS).

## RESULTS

### D-beta-hydroxybutyrate extends the lifespan of *C. elegans*

**Figure 1:**
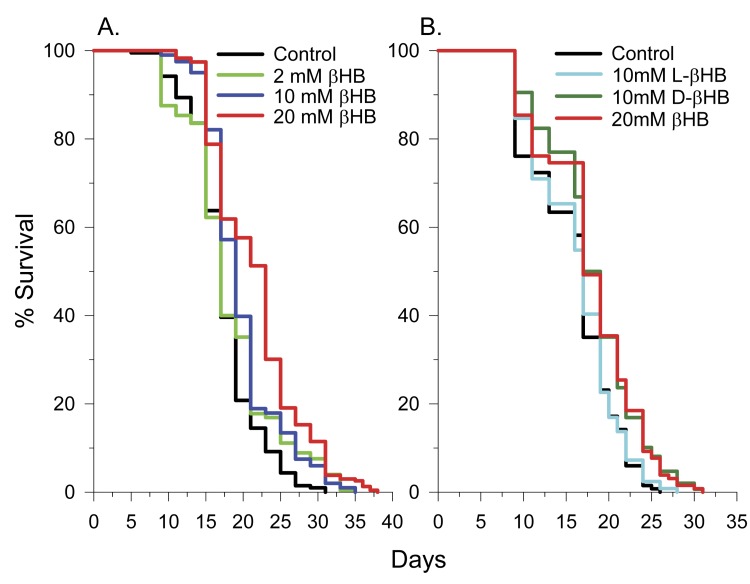
D-βHB extends the lifespan of N2 *C. elegans* worms. **(A)** Concentration dependency of βHB-mediated extension of lifespan. **(B)** D-βHB, but not L-βHB addition led to lifespan extension. When no D or L prefix is present, βHB refers to DL-βHB.

Addition of 2, 10, or 20 mM DL-beta-hydroxybutyrate (βHB) to the culture medium of *C. elegans* feeding on heat-killed E. coli increased lifespan with 20 mM having the greatest effect, increasing mean lifespan by 26%, from 17.2 to 21.7 days (Figure 1A). 50 mM and 100 mM concentrations decreased lifespan (Supplementary Table 1). Therefore a 20 mM concentration was used in further experiments. When *C. elegans* were fed live *E. coli*, 20 mM βHB only extended mean lifespan by 14%, from 16.0 to 18.3 days. This is likely due to catabolism of a portion of the βHB by the bacteria. To determine if the lifespan extension was due to D-βHB or L-βHB, we performed lifespan experiments with each isomer separately and found that only D-βHB addition resulted in lifespan extension (Figure 1B).

### βHB or butyrate individually, but not when combined, extend the lifespan of *C. elegans*

**Figure 2:**
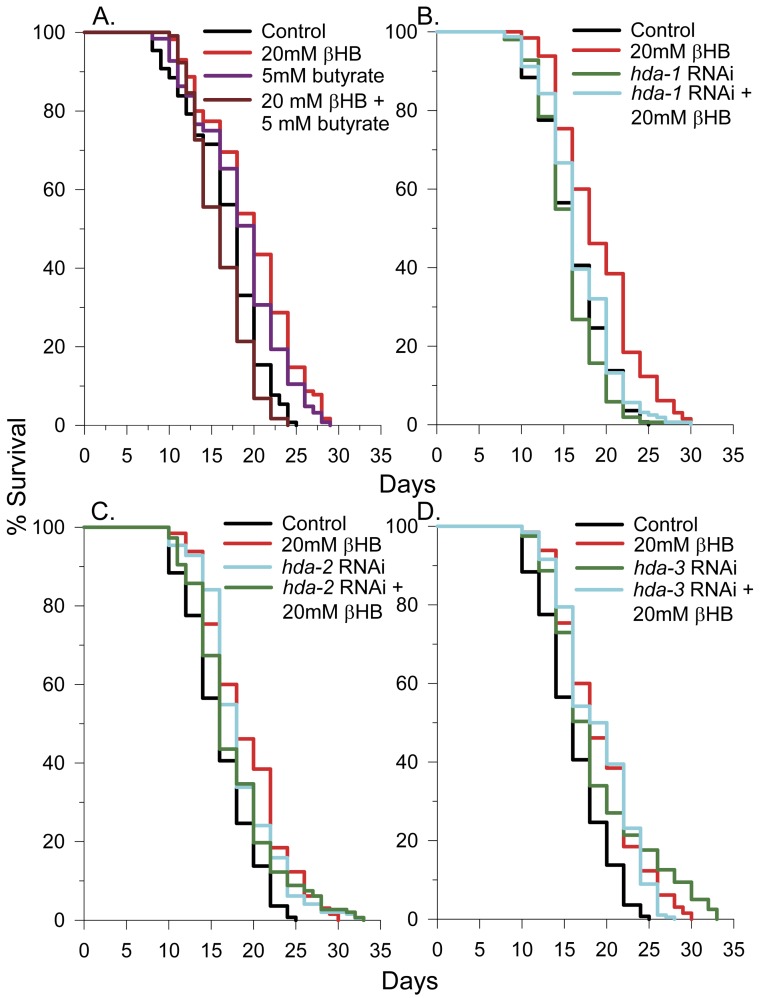
βHB-mediated HDAC inhibition plays a role in lifespan extension. **(A)** Survival of N2 worms in the presence of βHB, butyrate, or both compounds together. **(B)** Effects of *hda-1*, **(C)** *hda-2*, or **(D)** *hda-3* RNAi knockdown on *C. elegans* lifespan in the presence or absence of 20 mM βHB.

The histone deacetylase (HDAC) inhibitors sodium butyrate and valproic acid have been shown to extend lifespan in *C. elegans* [32, 33]. Since βHB has a similar chemical structure as butyrate and since βHB has been shown to inhibit class I and IIa histone deacetylases (HDACs 1, 3, and 4) in mammals with a K_i_ of 2-5 mM [13], we determined if βHB could further extend the lifespan of sodium butyrate treated worms. As shown in Figure 2A, and as previously found by others [33], sodium butyrate extended lifespan, but strikingly the combination of sodium butyrate and βHB led to a slightly decreased lifespan. This data is consistent with the possibility that βHB is functioning as an HDAC inhibitor as HDAC inhibitors such as valproic acid are known to cause decreased lifespan at higher concentrations in *C. elegans* ([32] and Supplementary Table 1). The combination of sodium butyrate and βHB likely has an additive inhibitory effect on HDAC activity, thereby decreasing lifespan. βHB addition also decreased the lifespan of worms treated with valproic acid (Supplementary Table 1), likely through a similar mechanism.

### Inhibition of HDA-2 and HDA-3 play a role in βHB-mediated lifespan extension

Many general HDAC inhibitors inhibit both class I and class II HDAC enzymes [13, 34]. The *C. elegans* genome has 3 class I HDACs, *hda-1*, *hda-2*, and *hda-3*. In addition there are 5 class II HDACs, *hda-4*, *hda-5*, *hda-6*, *hda-10*, and *hda-11*, with *hda-4* being the only member of class IIa [35]. To determine if HDAC inhibition plays a role in βHB-mediated lifespan extension, we knocked down the 3 class I HDACs in *C. elegans* by RNAi in the presence or absence of βHB and determined the effect on lifespan. RNAi knockdown of *hda-1* had no effect on lifespan and βHB addition extended lifespan, but to a lesser extent than in the absence of knockdown (Fig. 2B). However, RNAi knockdown of either *hda-2* (Fig. 2C) or *hda-3* (Fig. 2D) extended *C. elegans* lifespan, by 13% and 16% respectively, and prevented βHB from further extending lifespan. Therefore βHB likely extends lifespan in part through inhibition of *hda-2* and *hda-3*. We also performed lifespan analysis using *hda-2(ok1479)*, *hda-3(ok1991)*, *hda-4(ok518)*, and *hda-10(ok3311)* mutant worms (Supplementary Figure 1A-D). All of the HDAC mutant strains had roughly 30% decreased mean lifespans indicating that a partial knockdown, but not full knockout of HDA-2 or HDA-3 activity promotes lifespan extension. Consistent with the RNAi knockdown results, βHB addition did not extend the lifespan of the *hda-2* or *hda-3* mutant animals. However, βHB addition did lead to lifespan extension in the *hda-4* and *hda-10* mutants, suggesting βHB primarily extends lifespan through inhibiting the class I HDACs in *C. elegans*.

### F55E10.6 is likely a D-βHB inducible βHB dehydrogenase gene

There is no strong homolog of human mitochondrial BDH1 in *C. elegans*. However, a BLASTP search identified 4 candidate genes with 38-41% protein sequence identity with BDH1 (*dhs-2*, *dhs-20*, *dhs-16* (a 3-hydroxysteroid dehydrogenase [36]), and F55E10.6 (similar to human microsomal retinol dehydrogenase [37] and hydroxysteroid dehydrogenases [38]). DHS-2, DHS-16, and DHS-20 have been predicted to have a mitochondrial localization [39] like BDH1, however DHS-20 has also been predicted to have an ER localization [40] as has F55E10.6 [41]. Therefore, we knocked down each of the 4 candidates individually in the worms grown in the absence or presence of D-βHB and measured D-βHB dehydrogenase activity in the worm extracts. None of the knockdowns showed decreased basal D-βHB dehydrogenase activity.

**Figure 3:**
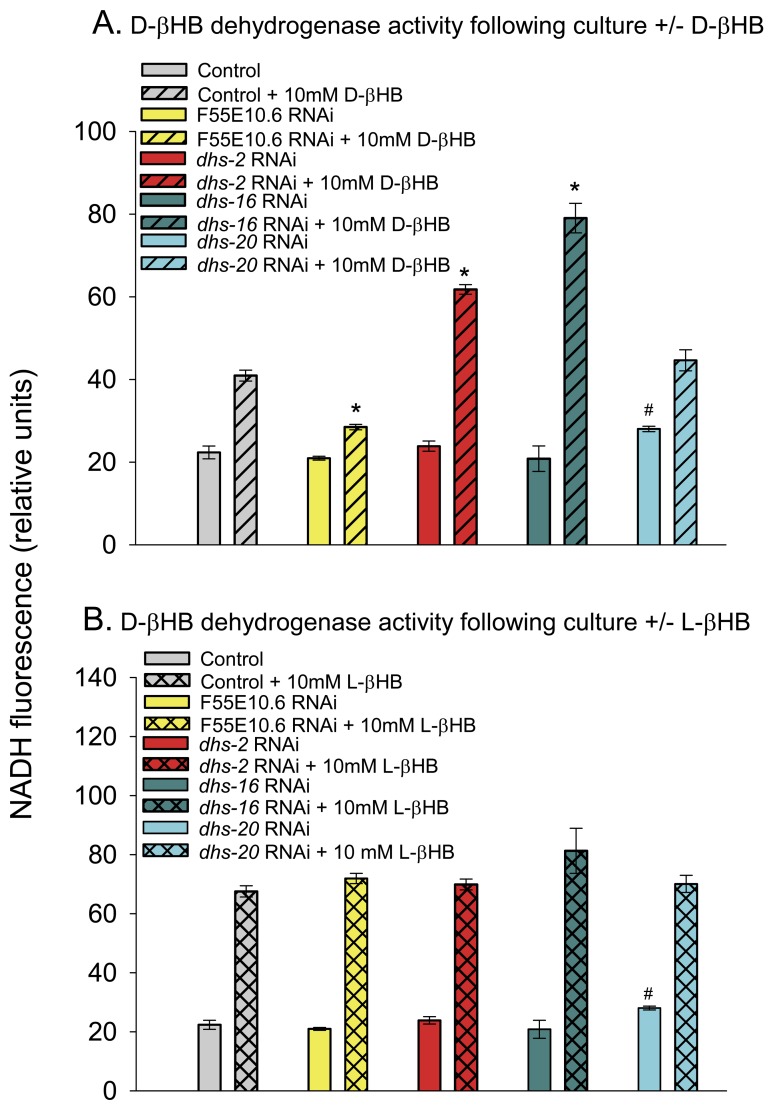
D-βHB dehydrogenase activity in worm extracts following RNAi-mediated gene knockdown. **(A)** D-βHB dehydrogenase activity following worm culture in the absence or presence of 10 mM D-βHB. Conditions in the legend refer to the culture conditions. The genes F55E10.6, *dhs-2*, *dhs-16*, or *dhs-20* were knocked down by RNAi feeding (* p < 0.05 compared to Control + 10 mM D-βHB; # p < 0.05 compared to Control). **(B)** D-βHB dehydrogenase activity following worm culture in the absence or presence of 10 mM L-βHB. The assay conditions were the same as panel A (# p < 0.05 compared to Control).

However, we found that adding D-βHB to the growth medium resulted in a 2-fold increase in D-βHB dehydrogenase activity and knockdown of F55E10.6 largely prevented this increased D-βHB dehydrogenase activity (Fig. 3A), suggesting that F55E10.6 likely encodes the D-βHB inducible D-βHB dehydrogenase activity. Knockdown of either *dhs-2* or *dhs-16* increased the ability of D-βHB to upregulate D-βHB dehydrogenase activity. We also found that adding L-βHB to the culture medium resulted in a roughly 3-fold increase in D-βHB dehydrogenase activity in the worm extracts that was independent of the expression of F55E10.6, *dhs-2*, *dhs-16*, or *dhs-20* (Figure 3B), suggesting L-βHB induces a separate D-βHB dehydrogenase enzyme. Consistent with this data, when worms were cultured with a racemic mixture of 20 mM βHB (DL-βHB) we found an almost additive 4.5-fold increase in D-βHB dehydrogenase activity (data not shown).

We also measured L-βHB dehydrogenase activity in the worm extracts (Supplementary Figure 2). There was roughly 5-fold lower basal L-βHB dehydrogenase activity than D-βHB dehydrogenase activity in the worm extracts (data not shown). Adding D-βHB to the culture medium yielded a roughly 50% increase in L-βHB dehydrogenase activity in control worm extracts that was almost completely blocked by RNAi knockdown of F55E10.6. Therefore, the F55E10.6 βHB dehydrogenase activity can likely utilize either D-βHB or L-βHB as substrates, but the activity with D-βHB appears roughly 10-fold higher than with L-βHB. Interestingly, knockdown of *dhs-2*, *dhs-16*, or *dhs-20* decreased basal L-βHB dehydrogenase activity in the extracts (Supplementary Figure 2). Determining whether any of these genes play a direct role in L-βHB metabolism, or if a gene encoding an enzyme with L-βHB dehydrogenase activity is down-regulated by knockdown of these genes awaits further investigation.

### F55E10.6 is required for βHB-mediated longevity

**Figure 4:**
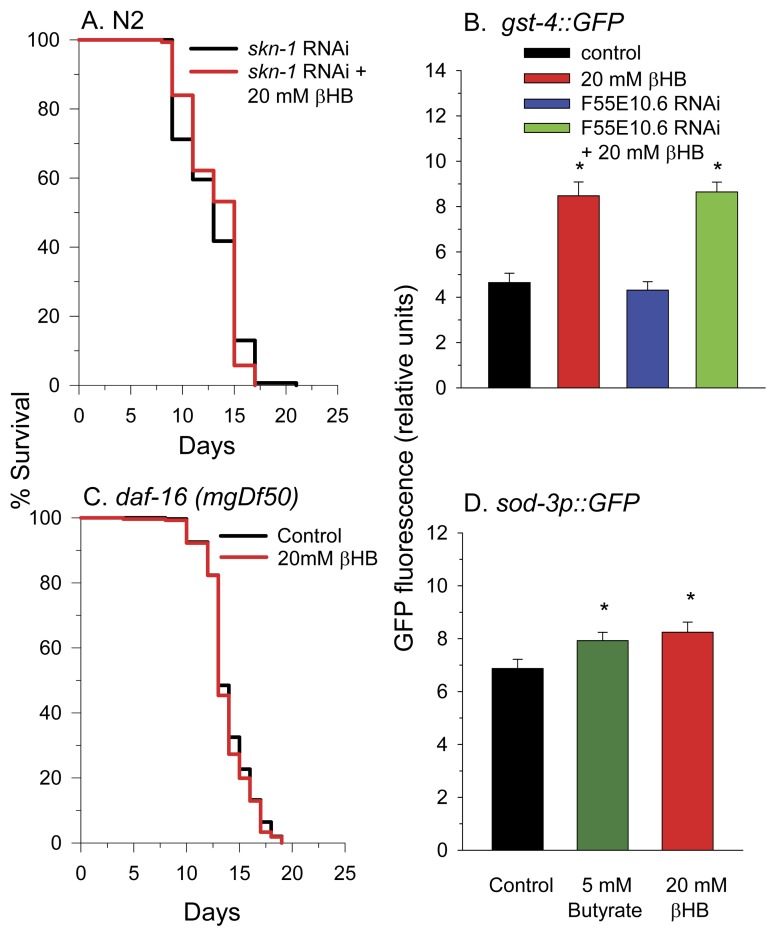
F55E10.6 is required for βHB-mediated lifespan extension, but not for βHB-induced oxygen consumption. (**A**) Treatment with βHB did not increase the lifespan of N2 worms fed RNAi to F55E10.6. **(B)** The addition of N-acetyl-L-cysteine (NAC) did not decrease the lifespan of βHB treated worms. **(C)** The effect of 20 mM βHB and RNAi knockdown of F55E10.6 on oxygen consumption (* p < 0.05 vs. untreated; # p < 0.05 vs. Control). **(D)** The effect of 20 mM βHB treatment on ATP levels in day 4 N2 worms (p = 0.202). Data are represented as mean +/− SEM.

To determine if βHB-mediated upregulation of F55E10.6 was essential for the effect of βHB on longevity, lifespan was monitored in worms in which expression of F55E10.6 was knocked down by RNAi feeding. RNAi knockdown of F55E10.6 increased lifespan by 7% and unexpectedly completely prevented lifespan extension induced by βHB supplementation (Figure 4A). F55E10.6 is a SKN-1 transcriptional target [42]. SKN-1 is a homolog of mammalian Nuclear factor (erythroid-derived 2)-like 2 (Nrf2) and a transcriptional regulator that induces the expression of genes involved in antioxidant defense and xenobiotic metabolism to promote longevity. Therefore, addition of βHB to the culture media activates SKN-1, which induces expression of F55E10.6. F55E10.6 could either metabolize βHB or metabolize another endogenous substrate leading to lifespan extension.

It is possible that metabolism of βHB, either dependent or independent of F55E10.6, is required for lifespan extension. This increased βHB metabolism may increase TCA cycle and electron transport chain (ETC) activity increasing ROS production, which has been shown to lead to lifespan extension in *C. elegans* [43]. Therefore we determined if administration of the antioxidant and glutathione precursor N-acetylcysteine (NAC) prevented the lifespan extension induced by βHB. As shown in Figure 4B, NAC by itself moderately increased lifespan, but NAC supplementation did not prevent lifespan extension mediated by βHB. Therefore, βHB is likely extending lifespan through a mechanism that does not require increased ROS production.

### Knockdown of F55E10.6 does not prevent the βHB-mediated increase in oxygen consumption

Since F55E10.6 expression was essential for βHB-mediated lifespan extension, we wished to determine if supplemented βHB was being utilized as a respiratory substrate by the worms and whether knocking down F55E10.6 would decrease βHB-induced respiratory metabolism. Therefore, we determined the effect of βHB supplementation on worm oxygen consumption (Figure 4C). βHB supplementation increased oxygen con-sumption by 2.3 fold indicating that βHB is being metabolized by the worms. Unexpectedly, we found that RNAi knockdown of F55E10.6 in the absence of βHB also increased oxygen consumption by around 2.3 fold, suggesting that F55E10.6 represses mitochondrial biogenesis or respiratory function. But RNAi knock-down of F55E10.6 did not decrease the βHB-mediated increase in oxygen consumption. The results suggest that the metabolism of βHB by F55E10.6 does not play a significant role in the use of βHB as an energy substrate for respiration, and so other mechanisms likely explain the requirement of F55E10.6 for lifespan extension.

We also determined the effect of βHB addition on worm ATP levels (Figure 4D). ATP levels were not significantly altered by βHB addition, although oxygen consumption rates increased suggesting that βHB either stimulates energy utilization pathways or decreases the coupling efficiency of oxidative phosphorylation. In this regard, we have previously shown that growth of *C. elegans* in the presence of the TCA cycle metabolites malate or fumarate resulted in a partial uncoupling of mitochondria [44].

### SKN-1 and DAF-16 are required for βHB-mediated longevity

**Figure 5:**
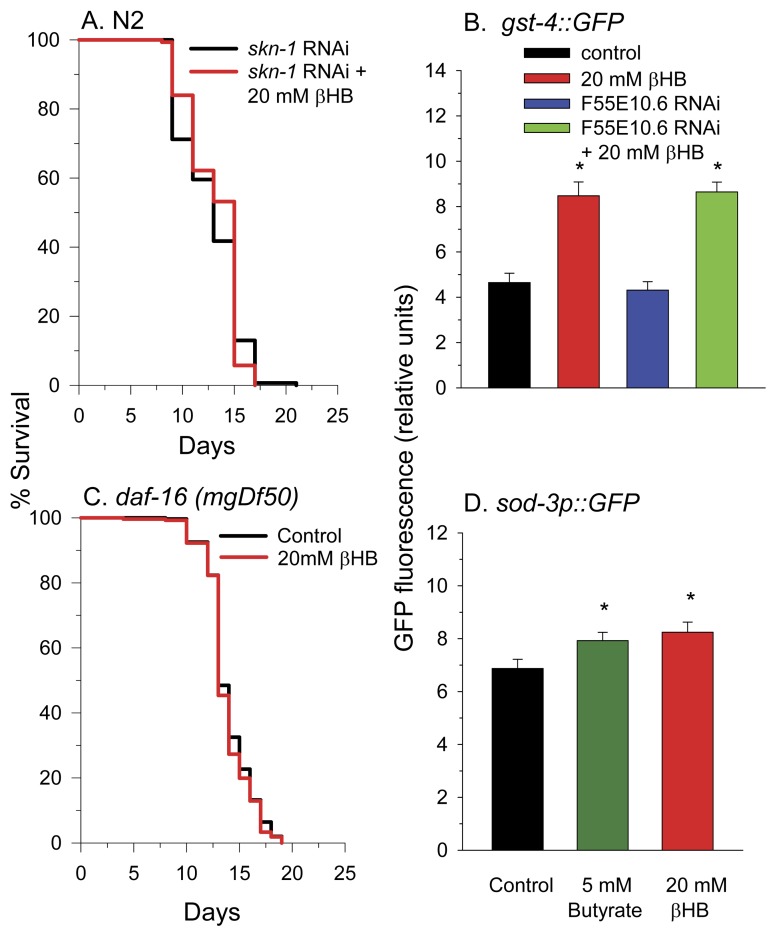
SKN-1 and DAF-16 are required for βHB-mediated lifespan extension. (**A**) βHB addition did not increase the lifespan of N2 worms fed RNAi to knockdown expression of *skn-1*. **(B)** βHB addition increased fluorescence of the *gst-4::gfp* SKN-1 reporter strain. Data are represented as mean +/− SEM (* p < 0.05). **(C)** βHB addition did not increase lifespan in *daf-16(mgDf50)* mutant worms. **(D)** βHB or butyrate increased fluorescence when administered to the *sod-3::gfp* DAF-16 reporter strain. Data are represented as mean +/− SEM (* p < 0.05).

To determine other molecular pathways through which βHB functions to extend lifespan, we performed lifespan experiments using worms deficient in common longevity pathways. In *C. elegans* the SKN-1 transcriptional activator is normally sequestered in the cytoplasm by WDR-23 and the DDB1/CUL4 ubiquitin ligase complex until the presence of specific xenobiotics or reactive oxygen species leads to a disruption of the interaction. This allows nuclear translocation of SKN-1 leading to the activation of a phase II detoxification transcriptional response and lifespan extension [45, 46]. We next determined if βHB extended the lifespan of worms in which SKN-1 levels were knocked down by RNAi feeding. Consistent with a role for SKN-1 and SKN-1 transcriptional targets such as F55E10.6 in βHB-mediated longevity, βHB did not extend the lifespan in these SKN-1 RNAi worms (Figure 5A). Additionally βHB was able to increase GFP fluorescence in the *gst-4*::*gfp* SKN-1 reporter strain of worms (Figure 5B) supporting this assertion. βHB was also able to induce expression of this reporter strain following knockdown of the F55E10.6 gene suggesting that F55E10.6 functions downstream of SKN-1 in the longevity pathway, as is expected for a SKN-1 transcriptional target.

Disruption of the DAF-2 insulin receptor signaling pathway is known to extend lifespan through activation of the DAF-16 transcriptional activator. DAF-16 is homologous to mammalian FOXO genes. βHB supplementation to worms homozygous for the *daf-16*(*mgDf50*) null allele did not lead to lifespan extension (Figure 5C). Furthermore, βHB supplementation slightly increased fluorescence of the *sod-3::gfp* DAF-16 reporter strain of worms (Figure 5D), supporting the ability of βHB treatment to activate DAF-16 activity for lifespan extension. Butyrate treatment also led to a similar small increase in GFP fluorescence of the *sod-3::gfp* worms suggesting a similar mechanism through which βHB and butyrate extend lifespan.

Another transcriptional regulator linked to longevity is hypoxia inducible factor-1 (HIF-1) [47]. We hypothesized that βHB catabolism would increase the concentration of TCA cycle metabolites that inhibit the alpha-ketoglutarate-dependent degradation of HIF-1 by the proteasome [48, 49] initiated by the EGL-9 prolyl hydroxylase [50]. However, we found that βHB supplementation did not increase fluorescence in the *nhr-57::gfp* reporter strain [47] for HIF-1 transcriptional activity (Supplementary Figure 3A). In addition supplementation with 10 mM pyruvate, or the TCA cycle metabolites citrate, succinate, fumarate, malate, or oxaloacetate also failed to induce GFP expression (Supplementary Figure 3B) suggesting that *C. elegans* HIF-1 may be regulated slightly differently than mammalian HIF-1.

### βHB increases thermotolerance

**Figure 6:**
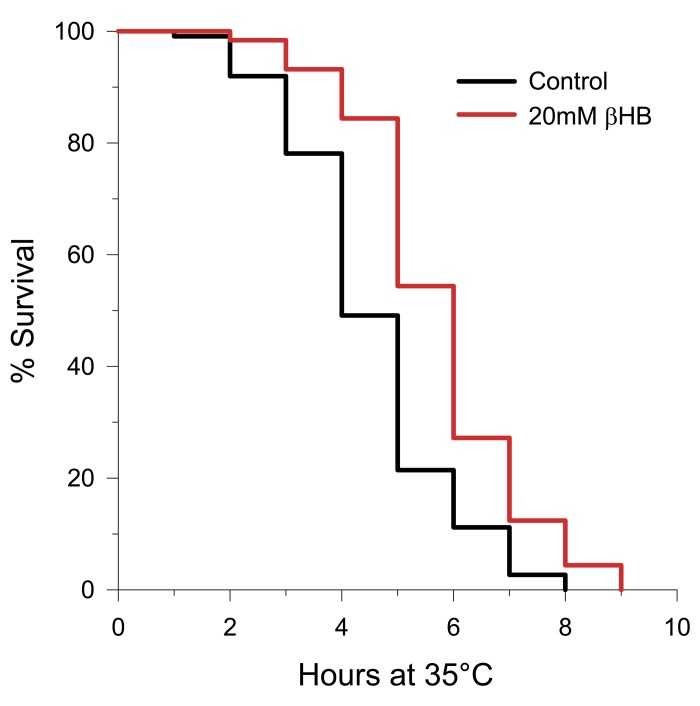
Treatment with βHB increases thermotolerance (log-rank *p* < 0.001) in N2 worms when upshifted from 20°C to 35°C. βHB mean survival time = 5.7 hours. Control mean survival time = 4.5 hours.

Since lifespan extension, and DAF-16 and SKN-1 activation in particular, has been linked to stress resistance, we determined the effect of βHB supplementation on thermotolerance in *C. elegans*. As shown in Figure 6, βHB administration extended the mean survival time of the worms after they were shifted to an elevated temperature by 22%. Due to the increased thermotolerance we hypothesized that heat shock proteins were induced by βHB supplementation. Therefore we monitored GFP fluorescence in 4 heat shock reporter strains of worms following βHB treatment (Supplementary Figure 4A-D). We used the strains *hsp-6::gfp* and *hsp-60::gfp* to monitor the mitochondrial unfolded protein response [51], *hsp-4::gfp* to monitor ER stress, and *hsp-16.2::gfp* to monitor heat shock factor-1 (HSF-1)-mediated gene expression [52]. βHB supplementation did not induce expression of any of these four reporter strains. Therefore βHB supplementation does not induce a broad heat shock response, even though thermo-tolerance was increased.

### Decreased protein synthesis rates likely contribute to βHB-mediated lifespan extension

**Figure 7:**
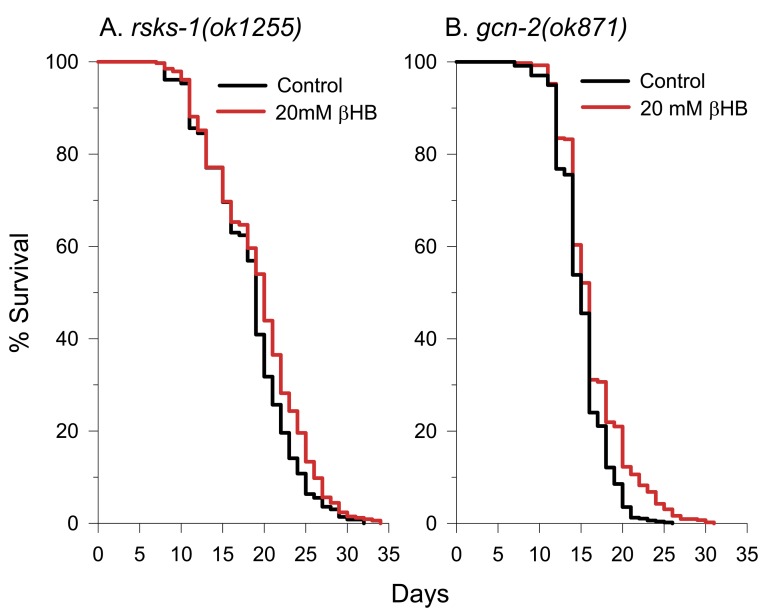
A decreased rate of protein synthesis contributes to βHB-mediated longevity. **(**A**)** βHB-mediated lifespan extension was blunted in *rsks-1(ok1255)* mutant worms. **(B)** βHB-mediated lifespan extension was also blunted in *gcn-2(ok871)* mutant worms.

Recently it has been recognized that several of the common longevity pathways converge to decrease the rate of translation initiation to extend lifespan [53, 54]. This can occur through several mechanisms including preventing phosphorylation of ribosomal protein S6 by S6 kinases of the TOR signaling pathway, blocking phosphorylation of eukaryotic initiation factor 4E (eIF4E) binding proteins (4E-BPs) by the TOR kinase, or by activation of general control nonderepressible 2 (GCN2) kinase. GCN2 activation can occur in the presence of uncharged tRNAs due to amino acid restriction [55] or during times when mitochondria produce high levels of reactive oxygen species [56]. *C. elegans* appears to lack close functional homologs of mammalian 4E-BPs [57] (although one distant homolog has been reported [58]), so they likely control the rate of translation initiation mainly through the p70 S6 kinase homolog RSKS-1 and the worm GCN2 homolog GCN-2. Therefore, we obtained the mutant strains *rsks-1(ok1255)* and *gcn-2(ok871)* and performed lifespan analysis in the absence or presence of βHB. The untreated lifespan of the *rsks-1* mutant was greater than that of the N2 control (Figure 7A) as expected, while the untreated lifespan of the *gcn-2* mutant was less than the N2 control (Fig. 7B). With either strain, we found that βHB-mediated lifespan extension was greatly blunted compared to the effect on the N2 control strain.

There was a 5% mean lifespan extension in the βHB-treated *rsks-1* mutant and a 8% mean lifespan extension in the βHB-treated *gcn-2* mutant compared to the 26% lifespan extension in the βHB-treated N2 control. Therefore, the ability to decrease translation rates through both the TOR/RSKS-1 and GCN-2 pathways likely allows for full βHB-mediated lifespan extension in the wild-type N2 animals.

### ETC Complex I function is needed for full βHB-mediated lifespan extension

**Figure 8:**
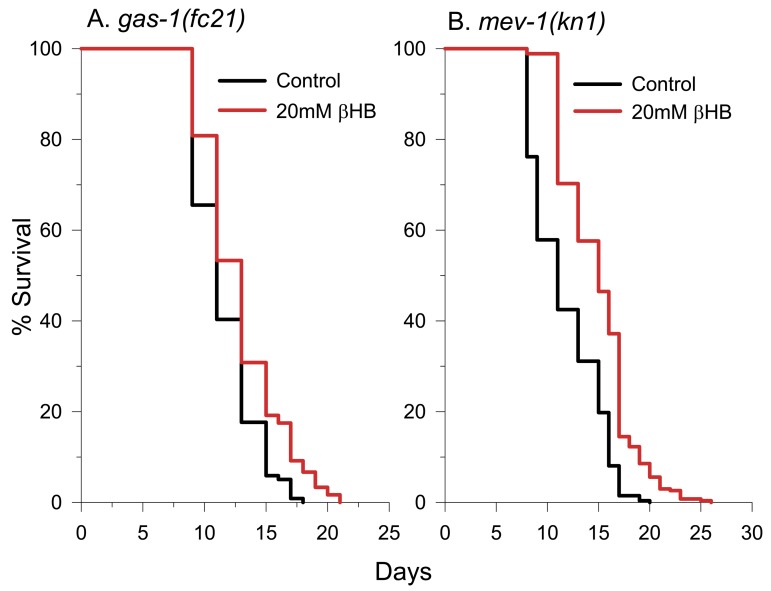
βHB extends the lifespan of short-lived mitochondrial ETC complex I and complex II mutants. **(A)** *gas-1(fc21)* survival in the absence and presence of βHB. **(B)** *mev-1(kn1)* survival in the absence and presence of βHB.

Following mitochondrial βHB dehydrogenase function, acetoacetate is converted to acetoacetyl-CoA with the concurrent conversion of succinyl-CoA to succinate as a byproduct of the succinyl-CoA: 3-ketoacid CoA transferase reaction. Because of this succinate production, it has been suggested that βHB protected a PD cell model by increasing mitochondrial ETC complex II (succinate dehydrogenase) activity, bypassing the ETC complex I deficits present in the disease [30]. To determine if normal mitochondrial ETC complex I or complex II activity is required for βHB-mediated lifespan extension, we determined the effect of βHB supplementation on the lifespan of short-lived complex I-defective *gas-1(fc-21)* mutants [59] (Figure 8A) and short-lived complex II defective *mev-1(kn1)* mutants [60] (Figure 8B). βHB extended the lifespan of the *gas-1* mutant by 11%, but not to the full 26% extent observed in wild-type worms. Therefore, normal ETC complex I function is necessary for the full effect of βHB on longevity. βHB supplementation fully extended the lifespan of *mev-1* mutants indicating that βHB does not require normal ETC complex II function to extend lifespan.

### βHB-mediated longevity requires AAK-2, SIR-2.1, CBP-1, and may occur in a similar manner as in DR

**Figure 9:**
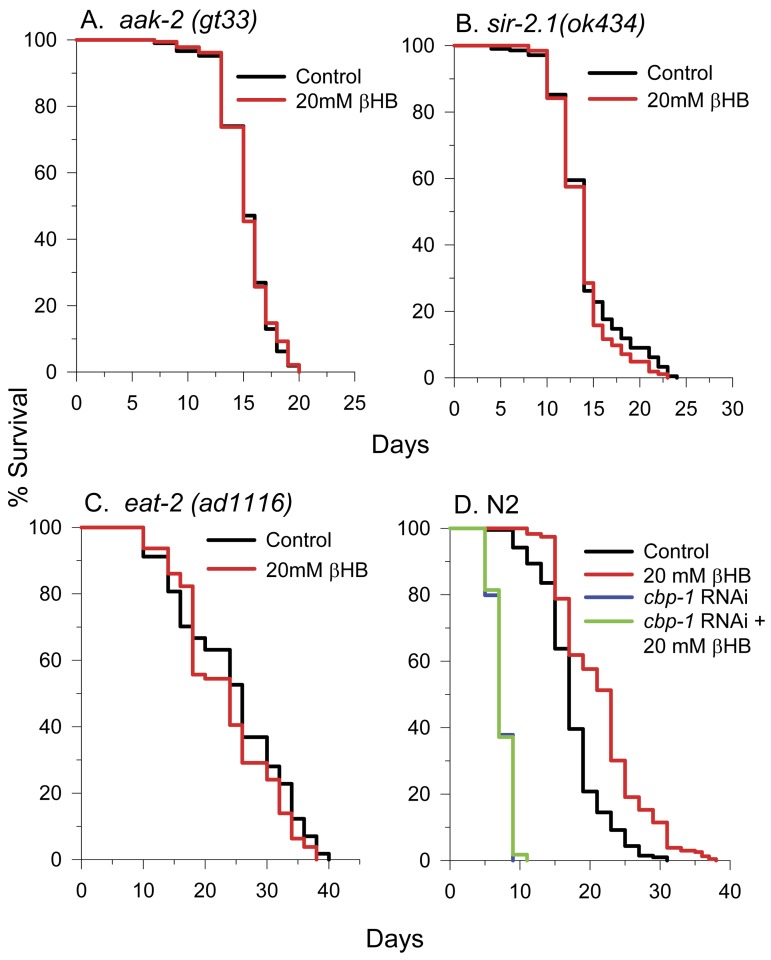
βHB extends lifespan in a similar manner as DR and requires AAK-2, SIR-2.1, and CBP-1. (**A**) βHB does not extend lifespan of AMPK mutant *aak-2(TG38)* worms, (**B**) *sir-2.1(ok434)* worms, or (**C)** *eat-2(ad1116)* worms. (**D**) Additionally, βHB does not extend the lifespan *cbp-1* RNAi knockdown N2 worms.

To identify if other important longevity regulators are required for βHB-mediated longevity, βHB was supplemented to AMP kinase (AMPK) *aak-2(TG38)* mutant worms (Figure 9A) and *sir-2.1(ok434)* NAD-dependent protein deacetylase mutant worms (Figure 9B) and lifespan was monitored. βHB addition did not extend the lifespan of either strain suggesting that both proteins play a role in βHB-mediated longevity. Since ketone body levels rise during caloric restriction (CR) in mammals and increased βHB levels may be responsible for some portion of the increased stress and disease resistance conferred by CR, we determined the effect of βHB supplementation on lifespan in the nematode *eat-2(ad1116)* model of dietary restriction (DR) in which pharyngeal pumping is slowed (Figure 9C). We found that treatment with βHB had no significant effect on the longevity of *eat-2* worms suggesting that βHB extended lifespan using some of the same downstream effectors activated in DR. The CREB binding protein-1 (CBP-1) transcriptional co-activator and protein acetyltransferase has been shown to be essential for DR-mediated longevity in *C. elegans* [33]. Therefore we determined the effect of βHB on lifespan in worms where *cbp-1* expression was knocked down by RNAi (Fig. 9D). Consistent with βHB extending lifespan in a manner similar to DR, knocking down *cbp-1* prevented lifespan extension induced by βHB treatment.

**Figure 10:**
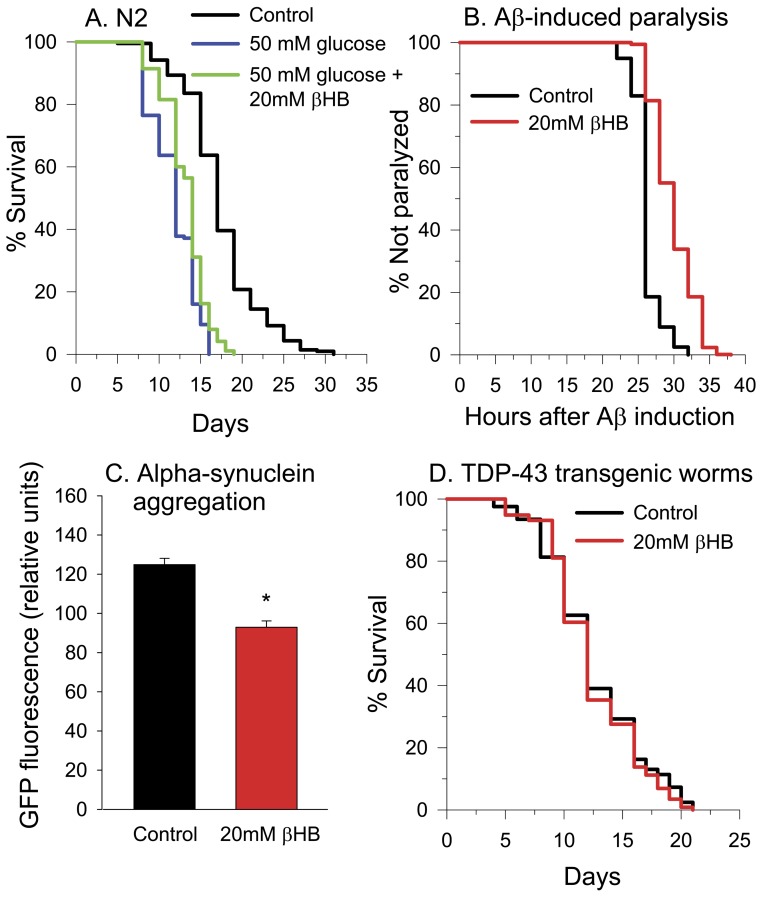
βHB protects against glucose toxicity and proteotoxicity. **(A)** Treatment with βHB partially protects against 50 mM glucose-induced reduction of lifespan in N2 worms. **(B)** Survival of the CL4176 strain of *C. elegans* expressing Aβ in muscle following temperature upshift. Treatment with βHB increases the time to paralysis (log-rank *p* < 0.001). βHB-treated mean lifespan = 29 hours, untreated control mean lifespan = 26 hours. The curves are generated from the results of six assays (*n* > 500 for both groups). **(C)** Treatment with βHB decreases α-synuclein-GFP aggregation in the NL5901 strain GFP fluorescence readings were taken on day 8 of worm lifespan. Data are represented as mean +/− SEM. (log-rank *p* < 0.001) **(D)** 20 mM βHB did not protect against the shortened lifespan induced by human TDP-43 overexpression when the worms were grown at 16°C.

Many of the same compounds that extend lifespan in *C. elegans* in a CBP-1 dependent manner also protect against glucose toxicity [61]. It's been shown that *C. elegans* shows a reduced lifespan when grown in a high glucose containing media [62-64]. When we grew worms in 50 mM glucose, lifespan was decreased by roughly 30% (Figure 10A). βHB supplementation to the glucose-containing media partially restored the lifespan, resulting in a lifespan reduction of only 21% compared to the non-glucose treated controls.

### βHB delays Aß-induced paralysis and decreases alpha-synuclein aggregation

We next performed experiments using a strain of worms engineered to express human AD-associated Aß peptide within body wall muscle upon temperature upshift from 16° to 25°C, which leads to paralysis of all worms by 32 hours after upshift [65]. Figure 10B shows the paralysis over time in these worms in the presence and absence of βHB treatment. βHB increased the mean paralysis time following Aß induction by 15%, from approximately 26 to 30 hours. Since βHB supplementation was beneficial in this model of proteotoxicity, we next determined the effects of βHB administration on a PD-model worm strain expressing human α-synuclein fused to yellow fluorescent protein (YFP) in the body wall muscle [66]. Alpha-synuclein protein is prone to aggregation and is the major protein constituent of Lewy bodies in PD brain [67]. YFP aggregation and fluorescence was decreased by 35% in worms treated with βHB for 8 days, indicating a protective decrease in the levels of alpha-synuclein aggregates (Figure 10C).

A ketogenic diet has been shown to delay loss of motor performance and loss of spinal cord motor neurons in the SOD1-G93A mouse model of amyotrophic lateral sclerosis (ALS) [68]. So lastly, we performed experiments using worms overexpressing human TDP-43 [69], which forms insoluble aggregates in the nervous system of patients with ALS and other neuro-degenerative disorders [70] and when expressed in the nervous system of worms [69]. TDP-43 expression caused a greatly reduced lifespan in *C. elegans* both when grown at 20°C and when grown at 16°C (Supplementary Table 1). 20 mM βHB supplementation was unable to prevent the reduction in lifespan (Figure 10D). Concentrations of βHB from 2 mM to 200 mM were also tested (Supplementary Table 1). Only 30 mM βHB was found to be effective at delaying toxicity and the increase in longevity at this concentration was only 5%.

## DISCUSSION

Administering βHB to *C. elegans* extended lifespan and delayed proteotoxicity and glucose toxicity. βHB extended *C. elegans* lifespan in a SIR-2.1 and AMPK-dependent manner that also required the stress-responsive transcription factors DAF-16 and SKN-1. Since βHB did not extend lifespan in *eat-2* pharyngeal pumping mutants, βHB likely acts as a dietary restriction mimetic, as previously hypothesized for its effects in mammals [71]. Even though protective effects of D-βHB on rodent disease models are known, this is the first report to identify D-βHB as a positive modulator of organismal longevity in wild-type animals. We also identified many of the signaling pathways and genes required for this effect. A key finding is that D-βHB-mediated lifespan extension requires SKN-1 and its transcriptional target F55E10.6, a short-chain dehydrogenase/reductase with βHB dehydrogenase activity, although inhibition of HDACS HDA-2 and HDA-3 are also required for the increased longevity.

### The role of F55E10.6 in βHB-mediated lifespan extension

The βHB dehydrogenase enzyme assay data suggest that F55E10.6 is a D-βHB-inducible βHB-dehydrogenase enzyme. However, D-βHB may not be the preferred physiological substrate for the enzyme or the substrate required for lifespan extension. Since knockdown of F55E10.6 did not affect the increased oxygen consumption following βHB supplementation, the enzyme does not likely possess a mitochondrial localization producing NADH for ETC complex I function. In this regard in addition to sharing homology with mitochondrial BDH1, F55E10.6 shares homology with microsomal retinol dehydrogenases [37] and microsomal hydroxysteroid dehydrogenases [38]. Due to these homologies and the role that SKN-1 plays in controlling the ER stress response [41], F55E10.6 has been putatively assigned an ER localization [41], although no signal peptide was found [40]. Key to its localization, F55E10.6 is predicted to have a transmembrane domain [40].

It is of interest that knockdown of F55E10.6, a SKN-1 target gene increased *C. elegans* oxygen consumption, suggesting that SKN-1 signaling may decrease mitochondrial biogenesis or function. Expression of F55E10.6 has been shown to decline with aging [72], likely due to the aging-related decline in activity of SKN-1 [42]. The mammalian SKN-1 homolog Nrf2 is also known to play a role in mitochondrial biogenesis. When overexpressed Nrf2 was shown to be a negative modulator of mitochondrial mass and membrane potential in a high throughput screen using C2C12 myoblast cells [73]. However, when upregulated under physiological conditions Nrf2 was found to be a positive regulator of mitochondrial biogenesis by inducing nuclear respiratory factor-1 (NRF-1) and peroxisome proliferator-activated receptor gamma coactivator 1-alpha (PGC-1α) expression in heart [74], liver [75], and lung [76].

### Full βHB-mediated lifespan extension requires mitochondrial ETC complex I function

βHB-induced lifespan extension was partially blocked in ETC complex I mutant worms and was unaffected in mitochondrial ETC complex II mutant worms. This likely suggests that the lifespan extension is driven partly by βHB metabolism-independent effects and partly by metabolism-dependent effects. Normal complex I activity may be needed to maintain a high NAD/NADH ratio beneficial for maximal lifespan extension [77]. In this regard, it has been shown that rotenone can induce a roughly 10-fold reduction in *C. elegans* respiration, but only a 2-fold reduction occurred in the presence of 10 mM βHB [78]. Therefore, βHB either stabilized complex I function in the presence of rotenone or stimulated complex II-dependent respiration to bypass this block of complex I function. In mammals βHB has been shown to stabilize and increase the efficiency of ETC complex I [68, 79]. The increased rate of NADH oxidation in the presence of βHB led to decreased ROS levels in mouse neocortical neurons following glutamate excitotoxicity [79]. βHB may also enhance complex I activity in worms, but it may not be able to fully do so in the *gas-1* mutants preventing full lifespan extension.

### Proposed mechanism for βHB-mediated lifespan extension

We propose 2 possible mechanisms for lifespan extension mediated by βHB supplementation. In the first mechanism (see Supplementary Figure 5), we propose that βHB directly inhibits HDACs to increase histone acetylation [14] causing gene expression changes leading to SKN–1 activation, independent of βHB catabolism. The metabolism-independent activation of SKN-1 is consistent with our previous data showing that stimulation of metabolism by supplementation of several TCA cycle metabolites did not activate SKN-1 transcriptional activity [44]. Next, SKN-1 activity induces expression of F55E10.6 [42, 45], required for proper execution of the SKN-1 longevity program. SKN-1 activation has been shown to repress expression of the insulin-like peptides DAF-28 and INS-39, decreasing DAF-2 insulin receptor signaling to activate DAF-16 [80]. βHB catabolism also likely increases the level of specific TCA cycle intermediates, which may contribute to the DAF-16-mediated lifespan extension. We and others have shown that supplementation of the TCA cycle meta-bolites fumarate, malate, and oxaloacetate activated nuclear translocation of DAF-16 to extend lifespan in an AMPK and SIR-2.1-dependent manner [44, 81]. Others have also found that the TCA cycle metabolite alpha-ketoglutarate extends lifespan through a TOR kinase-dependent mechanism [82]. Although this model is consistent with our data, is also possible that βHB-mediated HDAC inhibition causes a direct transcriptional upregulation of DAF-16, as βHB-mediated HDAC inhibition directly upregulates expression of the DAF-16 homolog FOXO3A in mammals [14].

In addition, βHB metabolism may increase acetyl-CoA levels that serve as a substrate for histone acetyl-transferases to increase histone acetylation [83], which could strengthen the effects of HDAC inhibition to extend lifespan. However, increased cytoplasmic acetyl-CoA levels have also been shown to inhibit autophagy [84], which could potentially dampen lifespan extension. However, the acetyl group from mitochondrial acetyl-CoA can be transferred to carnitine to form acetylcarnitine and exported from mitochondria to the nucleus, where acetyl-CoA is reformed and used for nuclear histone acetylation [85]. This mechanism may allow for increased histone acetylation without decreased rates of autophagy.

The second proposed model of how βHB may extend lifespan is through inhibition of the insulin signaling pathway. In mammals, it has been shown, contrary to expectations, that βHB administration or a ketogenic diet blocks the insulin signaling pathway in muscle leading to insulin resistance [86]. This adaptation likely evolved to allow the brain preferential use of the bloodstream glucose during starvation. However, a ketogenic diet has also been shown to be effective at lowering blood glucose in patients with type II diabetes due to the decreased carbohydrate intake [87]. In mouse studies, βHB administration yielded a 50% reduced phosphorylation and activity of Akt/protein kinase B downstream of the insulin receptor decreasing insulin signaling [86]. The mechanism for this βHB-mediated inhibition of Akt and the insulin signaling pathway was not fully elucidated, but it relied upon administration of D-βHB and not L-βHB, suggesting that mitochondrial metabolism of D-βHB may be involved. In *C. elegans*, inhibition or decreased expression of Akt or other proteins of the insulin signaling pathway has been shown to activate both DAF-16 and SKN-1 leading to lifespan extension [88], thereby providing a potential mechanism for the effect of βHB on longevity.

### βHB does not induce a broad heat shock response, but still increases thermotolerance

Although βHB did not induce expression of four specific heat shock proteins monitored (Supplementary Fig. 3), it did activate the DAF-16 and SKN-1 signaling pathways, which are both likely responsible for the increased thermotolerance observed following βHB treatment. Previous research has shown that RNAi knockdown of either *skn-1* or *daf-16* decreased thermo-tolerance [89]. DAF-16 is known to induce expression of several heat shock proteins including *hsp-12.6*, *sip-1*, and *hsp-16.1*, which may play a role in the increased thermotolerance. The factors that SKN-1 induce to confer thermotolerance are less clear, although SKN-1 function has been implicated in the induction of *hsp-4* expression and activation of the ER stress response [41]. However, we did not find βHB to induce *hsp-4::GFP* expression, but a positive trend was observed (*p* = 0.18).

### Neuroprotective effects of ketone bodies

In an AD cell model, βHB has been shown to protect hippocampal neurons from Aß toxicity [90]. The protection may have occurred through decreasing ROS levels as decreased ROS production is known to lower expression levels of beta-secretase (BACE1), a protease that contributes to toxic Aß generation [91]. This mechanism may be responsible for the ketogenic diet-induced reduction of Aß levels in a mouse model of AD [22]. The brain's ability to utilize glucose decreases in AD. To prevent deficits in brain ATP levels, βHB has been used as an alternative metabolic energy source for patients with AD [25]. Increased inflammation accompanies brain aging and may contribute to the development of AD. Increased levels of ketone bodies have been shown to reduce inflammation [71, 92] and this may result from increased mitochondrial efficiency and decreased ROS production [93].

PD is associated with aggregation of alpha-synuclein and death of dopaminergic neurons leading to motor decline. Mice treated with βHB showed partial protection against neurodegeneration and motor deficiency induced by MPTP [30]. Surprisingly, this was not due to increased NADH generation fueling complex I, but was described to be due to an increased supply of succinate, a substrate for ETC complex II [94]. As mentioned previously, the stimulation of complex II activity by βHB metabolism depends on the increased succinate produced as a byproduct of the mitochondrial succinyl-CoA: 3-ketoacid CoA transferase reaction. However, since βHB fully extended lifespan in complex II-defective *mev-1* mutants, it is unlikely that this mechanism plays a substantial role in βHB-mediated lifespan extension. The mechanism of βHB-mediated protection in PD models may be similar to the mechanism by which βHB supplementation increases lifespan in the complex I-defective *gas-1* worms.

### Study limitations and future directions

Although we were able to dissect many of the pathways through which βHB extends lifespan in *C. elegans*, many questions remain. For example, is the reason that D-βHB but not L- βHB extended lifespan due to the increased ability of D- βHB to be metabolized or due to the higher efficiency of D-βHB as an HDAC inhibitor? Is HDAC inhibition required for βHB-mediated upregulation of SKN-1 or DAF-16 activity? In addition to extending lifespan as shown here, RNAi knockdown of the class I HDAC *hda-3* was shown to protect against polyglutamine-mediated toxicity in a *C. elegans* Huntington's disease, while knockdown of most other HDACs increased toxicity [95]. Is HDAC inhibition or SKN-1 activity required for DAF-16 activation by βHB? Also is βHB catabolism required for βHB-mediated SKN-1 or DAF-16 activation? Furthermore, is the transcription factor PHA-4/FoxA, which is required for DR-mediated longevity [96], also required for βHB-mediated longevity? In this regard mammalian Foxa2 is known to induce expression of BDH1 [97]. Do βHB levels increase in DR worms and if so does this play a role in DR-mediated longevity? Lastly, are the same signaling pathways required for longevity also required for βHB-mediated protection in the *C. elegans* models of Aß and alpha-synuclein toxicity? Future experiments will provide answers to these questions and elucidate the molecular mechanisms responsible for the protective effects of βHB. This knowledge will allow for a broader use of βHB as a therapy for aging-related disorders.

## Conclusion

βHB treatment extended lifespan and protected against metabolic, proteotoxic and thermal stress in *C. elegans*. βHB-mediated lifespan extension occurred through induction of the DAF-16 and SKN-1 signaling pathways and was dependent upon βHB-mediated inhibition of HDACs HDA-2 and HDA-3. Our data support the hypothesis that βHB is a DR mimetic and that βHB treatment will likely be useful in the treatment of many human aging-associated disorders.

## MATERIALS AND METHODS

### *C. elegans* strains

*C. elegans* strains were obtained from the *Caenorhabditis* Genetics Center (CGC, University of Minnesota) and were cultured at 20°C in either liquid S media or NGM agar media [44] as indicated. Lifespan assays were performed using the following strains: N2 Bristol (wild-type), GR1307 [*daf-16(mgDf50*)], TG38 [*aak-2(gt33)*], DA1116 [*eat-2(ad1116)*], TK22 [*mev-1(kn1)*], CW152 [*gas-1(fc21)*], VC199 [*sir-2.1(ok434)*], RB1206 [*rsks-1(ok1255)*], RB967 [*gcn-2(ok871)*], VC983 [*hda-2(ok1479)*], RB1618 [*hda-3(ok1991)*], RB758 [*hda-4(ok518)*], and RB2416 [*hda-10(ok3311)*]. The following strains expressing GFP were used to monitor promoter activation: CL2166 [pAF15(*gst-4p::GFP::NLS*)], SJ4100 [*hsp-6::gfp(zcIs13*)], SJ4058 [*hsp-60::gfp(zcIs9*)], SJ4005 [*hsp-4::gfp(zcIs4)]*, ZG449 [*nhr-57p::GFP + unc-119(+)*], and CL2070 [*hsp-16-2::gfp(dvIs70*)]. The following strains were used as disease models: NL5901 [pkIs2386 (*unc-54p::alpha-synuclein::YFP + unc-119(+))*], CL4176 [*smg*-1^ts^ [*myo-3::Aβ_1–42_* *long 3′-UTR*]], and CL6049 [dvls62 (*snb-1::hTDP-43* + pCL26 (*mtl-2::GFP*)].

### Chemicals

Sodium DL-3-hydroxybutyric acid (βHB), sodium butyrate, valproic (2-propylpentanoic) acid, ethidium bromide, and potassium cyanide were purchased from Acros Organics. Sodium D-3-hydroxybutyric acid and L-3-hydroxybutyric acid were obtained from Sigma. 5-fluoro-2′-deoxyuridine (FUdR) was purchased from Research Products International Corp. and Biotang, Inc. Sodium hydroxide (Fisher Scientific) was added to metabolite stock solutions to obtain a pH of 7.0.

### Lifespan Analysis

*C. elegans* adults were bleached as previously described [44] to yield age-synchronized eggs in S-medium. Lifespan experiments were performed suspending eggs in liquid media in 3 μM transparent cell culture inserts (BD Falcon #353181) in 12-well microplates on an orbital shaker at 135 rotations/min at 20°C [98]. 1.3 mL of S-medium containing 9×10^9^ HT115 (DE3) *E. coli* per mL was placed in each well of a 12-well microplate. Then, bleach synchronized worm eggs were suspended at a concentration of 100–200 eggs/mL in the bacterial suspension in S-medium. Lastly, a cell culture insert was placed in each microplate well into which 0.25 mL of the egg/bacterial suspension (25-50 eggs) was placed (*n* = 3 wells per condition). Excluding RNAi lifespan experiments, bacteria were heat killed (using a Kendal model HB-S-23DHT ultrasonic cleaner) at 80°C for 60 minutes. Synchronized cultures of worms were cultured at 20°C and monitored until they reached adulthood (~72 h), at which time FUdR was added to a final concentration of 400 μM. Worm viability was scored every two days. Worms that did not respond to repeated stimulus were scored as dead and those that contained internally hatched larvae were excluded. The media containing bacteria in the wells of the microplate into which the culture inserts were submerged was removed and replaced with a new bacterial suspension every 3 days.

High glucose lifespan assays. We performed lifespans as described above with the addition 50 mM glucose to the culture medium. Animals were scored every day and inserts were transferred to fresh media every two days.

### RNAi treatment

The *E. coli* *skn-1*, *cbp-1, hda-1, hda-2, hda-3*, F55E10.6, *dhs-2*, *dhs-16*, and *dhs-20* clones from the Ahringer *C. elegans* RNAi library (Source BioScience LifeSciences), were grown for 16 hours and then given 1 mM IPTG to induce expression of the RNAi for 4 hours similarly as described in [99]. Lifespan experiments were performed as described above with the exception that live bacteria were used and the culture media in the microplate was replaced daily to replenish metabolite levels that may be partially depleted by bacterial metabolism.

### βHB dehydrogenase assays

D-βHB or L-βHB dehydrogenase activity was assayed as in [100], slightly modified from the original method published in [101]. The reaction mix contained 100 mM Tris-HCl pH 8.0, 10 mM MgSO_4_, 5 mm K^+^ EDTA, 400 mM hydrazine hydrate, 1 μ M rotenone, 10 mM NAD, 20 mm D-βHB or L-βHB, and 10 μ L of Halt protease inhibitor cocktail (Thermo Scientific). Worms were grown with or without D-βHB or L-βHB as indicated. On day 4 of development, worms were washed 3 times with M9 buffer to free them of bacteria and condensed to 50 worms per microliter. 1 mL of concentrated worms was subjected to 3 freeze-thaws cycles in liquid nitrogen. 50 μ L of each sample (~ 2,500 worms) was added to a clear bottomed 96-well microplate, followed by addition of 100 μ L of the reaction mix above. NADH fluorescence was measured kinetically for 20 minutes using 360/40 nm excitation and 460/40 nm emission filters (*n*=5) on a Biotek Synergy 2 microplate reader.

### Oxygen consumption measurements

N2 worms were grown in 12-well cell culture plates and fed HT115 (DE3) control or F55E10.6 RNAi knockdown-expressing *E. coli* as food for 4 days in the absence or presence of 20 mM βHB. Worms were washed 4 times using M9 buffer to free them of the bacteria and then resuspended in the culture media in which they were grown except without the bacteria. The average concentration of worms was obtained by taking ten 10 μL drops and counting the number of living worms in each drop. The volume of the culture was then adjusted to obtain a final concentration of 2 worms per μL. 300 μL of the worm suspension was then added to the chamber of a Clark oxygen electrode (MT200A chamber, Strathkelvin Instruments) and the respiration was monitored for 5 minutes. The respiratory rate was normalized to protein content by performing a protein assay on the worm suspension.

### ATP level measurements

ATP levels were measured using CellTiter-Glo reagent (Promega) on day 4 worms grown in the absence or presence of 20 mM βHB washed free of bacteria and then lysed by freeze-thaw as described in [44].

### GFP reporter strains

The GFP fluorescence of *C. elegans* populations was assayed using a Biotek Synergy 2 microplate reader. Strains were age synchronized and cultured in 12-well microplates as described above. At the L3 stage of larval development animals were treated with βHB or other compounds. Following 24 hours of treatment, worms were washed 3 times in S-medium and approximately 400 worms per 200 μL were added to a clear 96-well microplate and GFP fluorescence was measured using 485/20 nm excitation and 528/20 nm emission filters (*n*=10 per treatment group).

### Microscopy and quantification

Worms used for microscopy were anesthetized in M9 media containing 1mM levamisole and transferred to agar pads with glass coverslips and analyzed using an EVOS fluorescence microscope. Comparable results were established in the absence of levamisole (data not shown). Approximately 20 worms per condition were used and experiments were repeated at least three times (*n*=3). ImageJ™ software was used to quantify pixel densities.

### Thermotolerance assays

A synchronized population of N2 *C. elegans* eggs was placed on 20 mM βHB treated on non-treated NGM agar plates and allowed to hatch at 20°C. At the L4 larval stage of development animals were transferred to a 35°C incubator. Survival was scored as the number of animals responsive to gentle prodding with a worm pick. 224 βHB-treated and 250 non-treated worms were counted.

### Aß-mediated paralysis assays

Paralysis assays were carried out as outlined in [65]. Briefly, second generation synchronized gravid *C. elegans* strain CL4176 were placed on treated or untreated 6 cm NGM plates and allowed to lay eggs for 2 hours. After two hours, adults were removed and plates were placed in a 16°C incubator for 48 hours. Following 48 hours, plates were upshifted to a 25°C incubator. Scoring for paralyzed worms began 20 hours after upshift. Animals were scored for movement every two hours. Worms were considered paralyzed if they could not complete a full body movement after stimulation with a worm pick.

### Alpha-synuclein protein aggregation assays

Eggs from the NL5901 strain of *C. elegans* were treated with alkaline bleach, washed, and then placed in 12 well cell culture inserts as described above in the presence or absence of βHB. Following 3 days of treatment, FUdR was added to the inserts to prevent egg-laying and progeny development. Culture media in the microplates was changed every 3 days. On day 8 worms were washed 3 times with M9 media and placed on 1% agarose pads or immobilized with 10mM levamisole. Visualization of the number of inclusions expressing alpha-synuclein-YFP was captured using an EVOS fluorescence microscope. Foci larger than 2 μm^2^ were counted for each group (*n*=30) and measurements on inclusions were performed using NIH ImageJ™ software and the assay was completed at least 3 times similarly as performed in [102]. Statistical analysis was completed using GraphPad Prism software and calculation of statistical significance between various groups was carried out by Student's t-tests.

### TDP-43 lifespan assays

CL6049 [dvls62((*snb-1*::*hTDP-43* + pCL26(*mtl-2::GFP*))] second generation synchronized worms were placed on treated or untreated 6 cm NGM plates and allowed to lay eggs for 2 hours. After two hours, adults were removed and plates were placed in a 16°C incubator. At the L4 larval stage worms were transferred onto treated or untreated NGM plates with added 5-fluoro-2′-deoxyuridine (FUdR, 0.05 mg/mL) to inhibit egg-laying and growth of progeny. Worms were scored everyday by gentle touch with a platinum wire. Failure to respond to touch or move forward or backwards was scored as dead.

### Statistical Analysis

Kaplan-Meier survival analysis and log-rank tests were performed using Sigmaplot version 11.0. Student's t-tests were used in other analyses.

## SUPPLEMENTARY DATA


