# Supplementary material for: D-beta-hydroxybutyrate extends lifespan in C. elegans
Source: Aging (Albany NY). 2014 Aug 7;6(8):621–44. doi: 10.18632/aging.100683 (PMC4169858; doi:10.18632/aging.100683)
Supplement: Supplementary file 1 [file aging-06-621-s001.pdf]

## SUPPLEMENTARY DATA

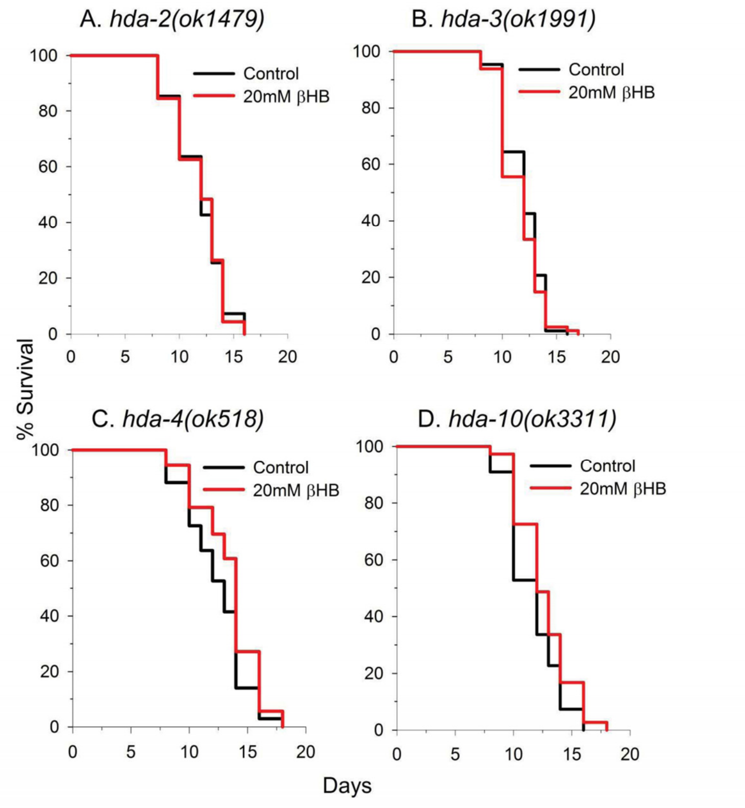


**Supplementary Figure 1.** The effect of βHB on lifespan in histone deacetylase mutants. βHB addition did not extend the lifespan of **(A)** hda-2(ok1479) mutant worms or **(B)** hda-3(ok1991) mutant worms. βHB addition extended the lifespan of **(C)** hda-4(ok518) mutant worms (log-rank p =0.001) and **(D)** hda-10(ok3311) mutant worms (log-rank p =0.002).

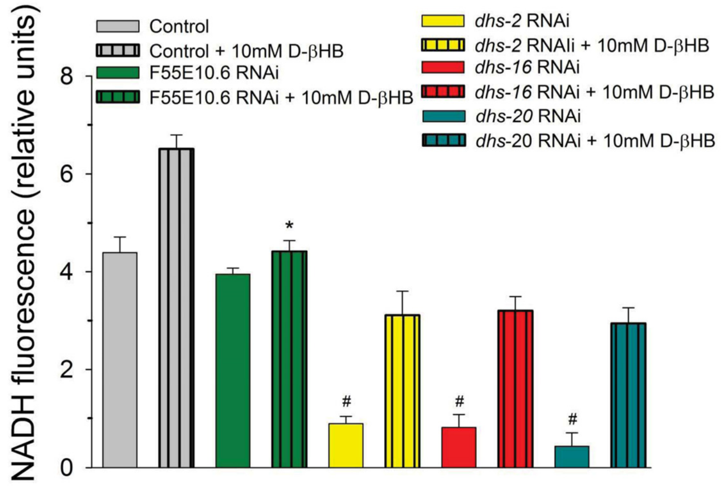


**Supplementary Figure 2.** L-βHB dehydrogenase activity in worm extracts grown in the absence or presence of 10 mM D-βHB. Knockdown of F55E10.6 almost completely prevented the increased L-βHB dehydrogenase activity induced by culturing with D-βHB (* $p < 0.05$ vs. (Control + 10mM D-βHB)). Knockdown of *dhs-2*, *dhs-16*, or *dhs-20* decreased endogenous L-βHB dehydrogenase activity in the extracts (# $p < 0.05$ vs. Control).

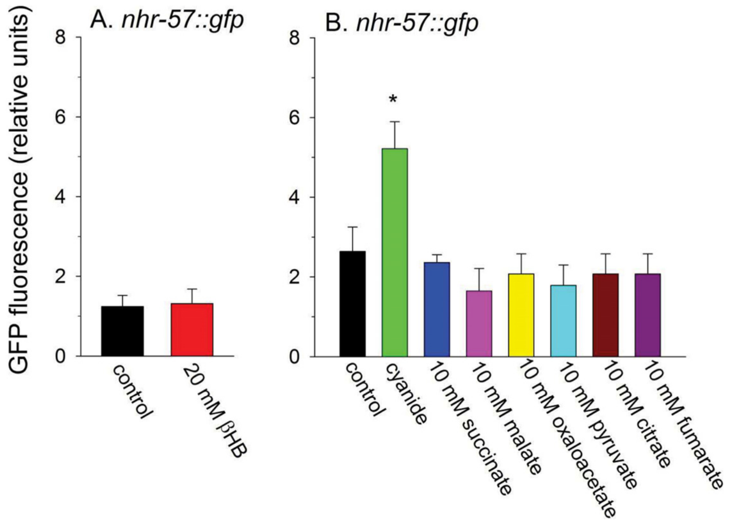


**Supplementary Figure 3.** βHB, pyruvate, or TCA cycle metabolites do not activate HIF-1 transcriptional activity. The effect of **(A)** βHB, **(B)** pyruvate, or TCA cycle metabolites on GFP fluorescence in nhr-57::gfp worms. 20 M potassium cyanide was used as a positive control. (* p < 0.05).

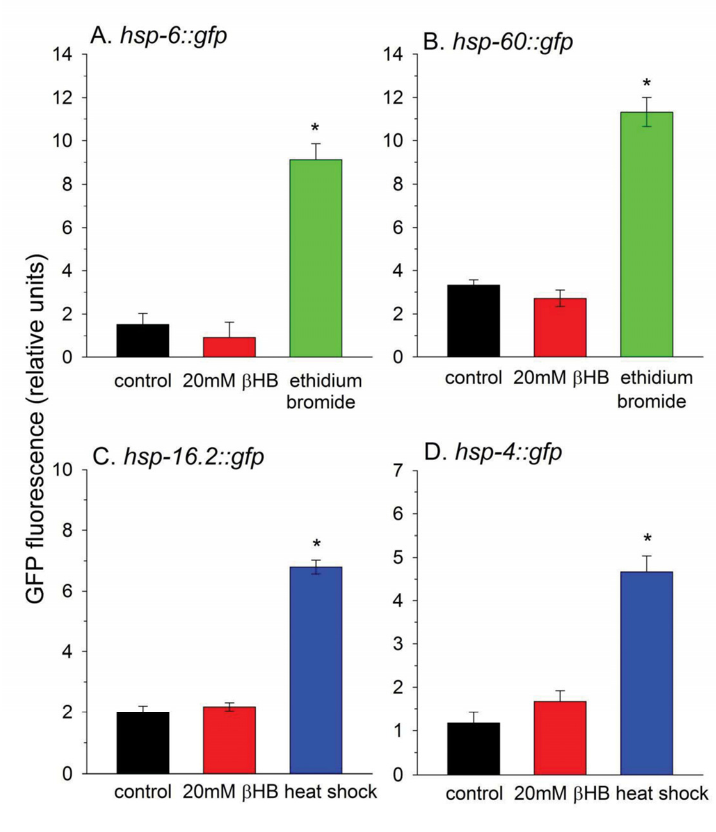


**Supplementary Figure 4.** βHB addition does not induce expression of several heat shock proteins. The effect of βHB addition on the fluorescence of **(A)** *hsp-6::gfp*, **(B)** *hsp-60::gfp*, **(C)** *hsp-16.2p::GFP*, or **(D)** *hsp-4::GFP* worms. For **(A)** and **(B)** 50 _g/ml ethidium bromide treatment for 2 days was used as a positive control. For **(C)** and **(D)** heat shock at 35°C for 2 hours was used as a positive control. (* $p < 0.05$).

Supplementary Table 1. Lifespan data

| Strain | RNAi | Treatment | % of untreated mean lifespan | % of N2 mean lifespan | *n* | # of worms | *p*-value |
|---|---|---|---|---|---|---|---|
| N2 | | 2 mM βHB | 105 | 105 | 2 | 224 | 0.024 |
| N2 | | 10 mM βHB | 113 | 113 | 2 | 201 | <0.001 |
| N2 | | 20 mM βHB | 126 | 126 | 6 | 586 | <0.001 |
| N2 | | 50 mM βHB | 81 | 81 | 1 | 100 | <0.001 |
| N2 | | 100 mM βHB | 69 | 69 | 1 | 100 | <0.001 |
| N2 | | 6 mM valproic acid | 93 | 93 | 2 | 118 | <0.001 |
| N2 | | 6 mM valproic acid + 20 mM βHB | 84 | 84 | 2 | 126 | <0.001 |
| N2 | | 5 mM butyrate | 110 | 110 | 2 | 124 | <0.001 |
| N2 | | 5 mM butyrate + 20 mM βHB | 95 | 95 | 2 | 117 | 0.023 |
| N2 | | 1 mM NAC + 20 mM βHB | 117 | 117 | 2 | 146 | <0.001 |
| N2 | | 1 mM NAC | 109 | 109 | 2 | 132 | 0.001 |
| N2 | | 50 mM glucose | 70 | 70 | 4 | 336 | <0.001 |
| N2 | | 50 mM glucose + 20 mM βHB | 79 | 79 | 4 | 362 | <0.001[a] |
| N2 | F55E10.6 RNAi | | | 107 | 3 | 375 | 0.008 |
| N2 | F55E10.6 RNAi | 20 mM βHB | 100 | | 3 | 403 | 0.527 |
| N2 | *skn-1* RNAi | | | 76 | 2 | 145 | <0.001 |
| N2 | *skn-1* RNAi | 20 mM βHB | 103 | | 2 | 156 | 0.557 |
| N2 | *cbp-1* RNAi | | | 44 | 2 | 118 | <0.001 |
| N2 | *cbp-1* RNAi | 20 mM βHB | 101 | | 2 | 112 | 0.803 |
| N2 | *hda-1* RNAi | | | 99 | 2 | 153 | 0.425 |
| N2 | *hda-1* RNAi | 20 mM βHB | 108 | | 2 | 159 | 0.002 |
| N2 | *hda-2* RNAi | | | 112 | 2 | 396 | <0.001 |
| N2 | *hda-2* RNAi | 20 mM βHB | 96 | | 2 | 362 | 0.018 |
| N2 | *hda-3* RNAi | | | 111 | 2 | 316 | <0.001 |
| N2 | *hda-3* RNAi | 20 mM βHB | 102 | | 2 | 380 | 0.648 |
| *hda-2(ok1479)* | | | | 69 | 1 | 110 | <0.001 |
| *hda-2(ok1479)* | | 20 mM βHB | 100 | | 1 | 91 | 0.98 |
| *hda-3(ok1991)* | | | | 69 | 1 | 87 | <0.001 |
| *hda-3(ok1991)* | | 20 mM βHB | 97 | | 1 | 81 | 0.29 |
| *hda-4(ok518)* | | | | 72 | 1 | 135 | <0.001 |
| *hda-4(ok518)* | | 20 mM βHB | 108 | | 1 | 125 | 0.001 |
| *hda-10(ok3311)* | | | | 67 | 1 | 110 | <0.001 |
| *hda-10(ok3311)* | | 20 mM βHB | 109 | | 1 | 113 | 0.002 |
| *daf-16(mgDf50)* | | | | 83 | 2 | 295 | <0.001 |
| *daf-16(mgDf50)* | | 20 mM βHB | 99 | | 2 | 270 | 0.361 |
| *aak-2(gt33)* | | | | 88 | 2 | 208 | <0.001 |
| *aak-2(gt33)* | | 20 mM βHB | 100 | | 2 | 184 | 0.793 |
| *sir-2.1(ok434)* | | | | 84 | 2 | 210 | <0.001 |
| *sir-2.1(ok434)* | | 20 mM βHB | 98 | | 2 | 265 | 0.113 |
| *eat-2(ad1116)* | | | | 146 | 2 | 114 | <0.001 |
| *eat-2(ad1116)* | | 20 mM βHB | 95 | | 2 | 157 | 0.114 |

| | | | | | | | |
|---|---|---|---|---|---|---|---|
| *gas-1(fc21)* | | | | 69 | 2 | 119 | <0.001 |
| *gas-1(fc21)* | | 20 mM βHB | 111 | | 2 | 120 | <0.001 |
| *mev-1(kn1)* | | | | 70 | 2 | 273 | <0.001 |
| *mev-1(kn1)* | | 20 mM βHB | 127 | | 2 | 269 | <0.001 |
| *rsks-1(ok1255)* | | | | 106 | 3 | 362 | <0.001 |
| *rsks-1(ok1255)* | | 20 mM βHB | 105 | | 3 | 337 | 0.002 |
| *gcn-2(ok871)* | | | | 88 | 3 | 479 | <0.001 |
| *gcn-2(ok871)* | | 20 mM βHB | 108 | | 3 | 428 | <0.001 |
| CL6049 *(16°C) snb-1::TDP-43* | | | | 57 | 1 | 123 | <0.001 |
| CL6049 *(16°C) snb-1::TDP-43* | | 20 mM βHB | 99 | | 1 | 114 | 0.413 |
| CL6049 *(16°C) snb-1::TDP-43* | | 30 mm βHB | 105 | | 1 | 117 | 0.006 |
| CL6049 *snb-1::TDP-43* | | | | 61 | 1 | 90 | <0.001 |
| CL6049 *snb-1::TDP-43* | | 50 mM βHB | 101 | | 1 | 93 | 0.951 |
| CL6049 *snb-1::TDP-43* | | 100 mM βHB | 103 | | 1 | 53 | 0.791 |

[a]Compared to 50 mM glucose treated worms or untreated worms.

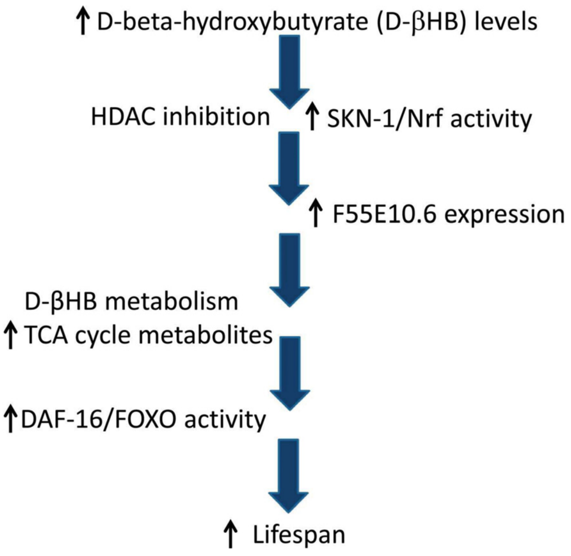


**Supplementary Figure 5.** One possible mechanism through which βHB may extend lifespan in *C. elegans.*
